# The significance of glucose, insulin and potassium for immunology and oncology: a new model of immunity

**DOI:** 10.1186/1476-8518-3-5

**Published:** 2005-08-19

**Authors:** Albert F Hill, William J Polvino, Darcy B Wilson

**Affiliations:** 1 Hill Medical, LLC, 1755 Monaco Parkway, Denver, CO. 80220-1644, USA; 2 Rejuvenon Corporation, 621 Shrewsbury Ave., Shrewsbury NJ, 07702, USA; 3 Torrey Pines Institute for Molecular Studies, 3550 General Atomics Court, San Diego, CA, 92121-1122. USA

## Abstract

**Background:**

A recent development in critical care medicine makes it urgent that research into the effect of hormones on immunity be pursued aggressively. Studies have demonstrated a large reduction in mortality as a result of infusion with glucose, insulin and potassium. Our work in the oncology setting has led us to propose that the principal reason for such an effect is that GIK stimulates lymphocytes to proliferate and attack pathogens, sparing the patient the stress of infection. That suggestion is based on a new model of immunity that describes the effect of hormones on lymphocytes. We hypothesized that the application of glucose, insulin, thyroid and potassium would awaken inert tumor infiltrating lymphocytes to destroy the tumor.

**Methods:**

The antitumor effect of a thyroxine, glucose, insulin, and potassium (TGIK) combination was studied in a series of controlled experiments in murine models of tumor progression to assess the biologic activity of the formulation, the effect of route of administration, the effect on tumor type, and the requirement for insulin in the TGIK formulation.

**Results:**

Melanoma and colon tumors inoculated with TGIK were significantly reduced in size or retarded in growth compared to controls injected with saline. I.P. and I.M. injections showed that the formulation had no effect systemically at the doses administered.

**Conclusion:**

We conclude that TGIK has anti-tumor activity when administered intratumorally, probably by stimulating lymphocytes to attack tumors. This is similar to the effect of GIK on reducing sepsis in critical care patients. We suggest that when GIK is administered exogenously, it restores immune competence to the critically ill or cancer patient and causes destruction of pathogens or tumors, while endogenous resources are devoted to repair. This implies that hormonal therapy may be useful in treating various other pathologies involving immune suppression, as well as malignancies. We also propose research that could bring resolution of the controversy over mechanism and point the way to new therapeutic strategies for numerous diseases including chronic infections and auto-immune diseases.

## Background

In a turnaround from the usual laboratory research-to-clinical usage sequence, critical care has become the focus for one of the most interesting developments in medicine: the use of glucose, insulin and potassium (GIK) in treating the critically ill. Van den Berghe *et al*., in a landmark study, demonstrated a 46% reduction in mortality [1]. Krinsley, with a less aggressive protocol, produced similar results [2]. Since the greatest reduction was in deaths due to multiple-organ failure with a septic focus, the implications for immunology could be significant. Steinman and Mellman recently made a strong case that only research in human beings can advance our understanding of the human immune system [3]. The discoveries involved in the use of GIK supports that. It has been known for years that lymphocytes have receptors for numerous hormones and neurotransmitters, but that fact is seldom incorporated into models of the immune system [4]. Impressive progress has been made in many areas of immunology, particularly in the ways cells communicate with and affect each other. Now the success of GIK suggests that a hormone, insulin, strongly enhances the immune response. The time has come to examine more closely the role endocrine hormones play in regulating immunity. Deciphering the mechanism of GIK is crucial, not only for critical care, but also for a better understanding of immune response mechanisms.

Van den Berghe first speculated that strict glycemic control provided the beneficial effect of GIK; more recently she has suggested that the most important benefit may be from the "powerful anti-inflammatory effect" of insulin. Hyperglycemia can contribute to inflammation, and insulin has anti-inflammatory properties (e. g. inhibiting production of tumor necrosis factor-alpha and super-oxide radicals, macrophage migration inhibitory factor) [5-7], and TNFα and IL-1 have been shown to depress myocardial function in a dose-dependent fashion [8]. Still, it is unlikely that inflammation is producing the deleterious effects in the critically ill. IL-1, which is so central in inflammation, is known to suppress the expression of insulin-like growth factor-1 [9]. Yet Van den Berghe found levels of IGF-1 to be high in her patients, particularly those near death. Also, inflammation is an early, indispensable part of a robust immune response. Without phagocytes ingesting pathogens, presenting antigen and releasing cytokines, lymphocytes would not become activated effector cells. Infection would rage unabated. To be maximally effective, the immune sequence must move from the inflammatory to the acquired, lymphocytic phase. A remarkable aspect of immunity is the way the body selects and produces the right response to a given challenge. If an infection is contained, inflammation will be chosen as the appropriate defense, and the cytokines released will actually restrain the expansion of lymphocyte clones. If the response must proceed from inflammation to the adaptive phase, cytokines from damaged tissue, macrophages and dendritic cells instruct CD4 cells to become T_h_1 or T_h_2 cells, according to which kind of lymphocyte, CTL or B cell, is needed. Cytokines released by those cells then restrain inflammation but advance the lymphocyte response. For example, Interleukin 6, which is both pro- and anti-inflammatory at times, promotes proliferation of CD8 cells, and suppresses inflammation by down-regulating TNF-α. IL-1 and chemokine expression [10]. Interleukin 4, produced by T_h_2 cells also suppresses the production of Il-1, TNF-α, and chemokines [11]. Interleukin 10, another anti-inflammatory T_h_2 cytokine, down-regulates synthesis of IL-1, IFN-γ, IL-2, TNF-α [12].

Cytokines also have a powerful effect on metabolism. Il-6 and TNF-α cause loss of skeletal muscle protein and lean tissue wasting, insulin resistance, increased glucogenesis, increased lipolysis in adipose tissue, and development of cachexia [13]. These changes provide a rich substrate for use by dividing immune cells. The body will also increase the secretion of endocrine hormones that will further enhance the expansion of the cells needed for the particular challenge. For example, insulin will suppress inflammation but, as we shall see, it will also stimulate a rapid expansion of lymphocyte clones. It has been known for decades that following trauma, hyperglycemia without increased insulin secretion occurs [14-16], and that the degree of hyperglycemia is correlated with the severity of the injury [17,18]. We therefore suggest that hyperglycemia is the normal response of the body as it tries to make nutrients available for the repair of damaged tissues. If, after a trauma or inflammation, systemic infection occurs, insulin will rise as the body supports the expansion of lymphocyte clones. (see below)

Years ago it was discovered and confirmed that insulin powerfully enhances the capacity of cytotoxic T lymphocytes *in vitro *to kill targets bearing the sensitizing antigen [19] and to do so in a dose-dependent manner within the physiological range [20,21]. While circulating quiescent lymphocytes have no detectable insulin receptors, once they have received antigenic challenge, they acquire approximately 6,000 per cell [22-26]. Since acquisition of these receptors is an early event in cellular transformation, it seems probable that the emergent insulin receptors are a prerequisite for, rather than a consequence of cell enlargement and subsequent cell division [27-29]. Insulin is, therefore, an immuno-regulatory hormone [30].

The effect of insulin on lymphocytes becomes significant when seen as part of the profile of events when a body is challenged by infection. More than twenty years ago Beisel mapped the response of the body to an infectious challenge [31]. He showed that the first detectable response was phagocytic activity, followed by increased secretion of glucocorticoids and growth hormone, deiodination of thyroxine, secretion of acute phase proteins, carbohydrate intolerance, increased secretion of aldosterone and ADH and eventually an increased secretion of thyroxine. One of his many contributions included the discovery that IL-1 (then called Leukocyte Endogenous Mediator) also acts as a hormone, stimulating uptake of amino acids and increasing synthesis of acute phase reactants [32]. Beutler *et al*., pointed out that the inflammatory cytokine, Tumor Necrosis Factor (TNF), once called cachectin, suppresses lipoprotein lipase, and causes peripheral tissues to lose nutrients [33]. The net effect of this is to mobilize energy reserves and make them available to dividing inflammatory and immune cells [34].

Rayfield and associates studied the effect of acute endotoxemia on volunteers and showed that during the febrile phase of an infection *insulin increases to three times basal levels *(35 ± 5 μU/ml) and, paradoxically, glucagon increases to five times normal [35]. Other investigators have confirmed this threefold rise in insulin during an infection [36,37]. In this "Infectious Mode," lymphocytes produce insulin receptors at the very time the hormone is rising in the blood, and are able to bind it and acquire glucose. But if insulin is low in the blood, even lymphocytes displaying insulin receptors cannot activate. The rise in glucagon assures a supply of glucose for the expanding clone of lymphocytes. They are then able to pump ions, which, we shall see, is the *sine qua non *of full lymphocyte activation. Insulin and thyroid increase the activity of the sodium potassium pump [38].

The endocrine mix produced *after *an infection or trauma, when the body is repairing damaged tissues, is quite different. In this "Healing Mode," insulin levels drop to normal or lower levels, counter-regulatory hormones such as growth hormone and cortisol continue to be high [39], and the liver increases production of insulin-like-growth-factor-1 (IGF-1). IGF-1 and autocrine growth factors enable the dividing reparative tissues to acquire nutrients from the blood even as peripheral tissues are starved. Thus, the body cannibalizes peripheral tissues for the sake of repairing the wound [40]. This endocrine mix is powerfully immuno-suppressive, as all the body's resources are devoted to repair. The degree of hyperglycemia and IGF-1 are indices of the degree of injury. Van den Berghe found that rising IGF-1 levels predict mortality accurately [41].

When a patient is critically ill, the body responds quickly with "...a highly coordinated and powerful acute phase reaction, whereby the immune system is switched from the adaptive mode of response to the amplification of natural immune mechanisms." "The increased serum level of cytokines and the array of neuroendocrine changes lead to fever, catabolism and to the suppression of the T lymphocyte-dependent adaptive immune system. At the same time natural immune mechanisms are amplified" [42]. If pathogens are present, lymphocytes will later enter the battle. However, if the injury itself is life-threatening, we propose the body will not proceed to the next phase of supporting the expansion of lymphocyte clones but instead will move into the Healing Mode, described above, so that all bodily resources can be devoted to repair of damaged tissues. In this environment, inflammation can continue, sometimes with destructive force, but there can be no significant involvement by lymphocytes because insulin is too low. Immune competence in the seriously wounded patient is severely reduced.

Therefore we propose that it is not inflammation *per se *that harms the critically ill patient; it is the incapacity of the body to complete the immune sequence and protect itself against infection. Exogenous GIK enables inert lymphocytes to proliferate and perform cytotoxic tasks, even as endogenous resources are devoted to repair of tissues.

As evidence of how GIK stimulates immunity *in vivo*, we offer this. A few years ago, we developed a new model of immunity that incorporates the effects of endocrine hormones and neurotransmitters on lymphocytes. Lymphocytes are chemotactically attracted to a tumor and actually invade it (TILs), but they do little damage. Some of that failure is due to the immunosuppressive effect of autocrine growth factors produced by the tumor (*e.g*. Transforming Growth Factor beta (TGFβ) [43]. But there is more to the problem: in a tumor-bearing animal, the suppression is systemic [44].

We proposed that the brain of a tumor-bearing animal is "deceived" by growth factors released by the tumor. The brain treats the malignancy as if it were a healing wound and commands an endocrine mix to support growth and suppress immunity. The mix features decreased levels of insulin and increased amounts of counter-regulatory hormones. Peripheral tissues become insulin resistant and lose nutrients into the blood, sometimes producing hyperglycemia and eventually the familiar cachexia of the cancer patient. The dividing tumor cells (like those involved in repair of damaged tissue) can utilize the materials lost by peripheral tissues, because they produce autocrine growth factors [45]. And, again, the liver increases production of IGF-1. As does a healing wound, the tumor cannibalizes the body for the materials it needs to grow [46].

As mentioned above, when the lymphocyte is deprived of high levels of insulin, it cannot acquire glucose and the sodium/potassium pump cannot restore ionic integrity. With its stores of potassium reduced, the lymphocyte cannot complete its enzymatic actions and transform or proliferate. This effect on the sodium/potassium pump is crucial; at every point in a lymphocyte's activation and proliferation, and in the performance of its function, the cell loses its surface charge, ion channels open, potassium escapes and sodium rushes in, down the electro-chemical gradient [47-49]. Before the lymphocyte can proceed in its cycle, it must replenish stores of potassium [50-52]. If it is 20% deficient in that ion, it cannot continue its cycle of mitosis or perform its function [53]. Yet cancer patients are as much as 40% deficient in total body potassium [54]. It is also significant that when insulin is administered i.v. and blood levels rise to three times normal, potassium moves into the cells [55,56].

We hypothesized that if a cancer patient were to be administered thyroid and insulin (to stimulate the sodium/potassium pump), glucose and potassium (TGIK), all in quantities to mimic those reached during an infectious challenge, inert lymphocytes would activate and destroy a tumor.

Presented here are partial results from controlled studies with mice. At the request of investors, Hill Medical has not heretofore published any results.

## Methods

Melanoma cells were injected into mice, and when the tumors became palpable they were inoculated with TGIK or saline solution. In another study mice were injected with only part of the combination to determine if insulin were necessary, or if irritation by potassium were producing the results. In further experiments the formula was tested by injecting I.M. and I.P. Still another tested the effect of the formulation on colon cancer.

### Experiment 1

**Figure 1:**
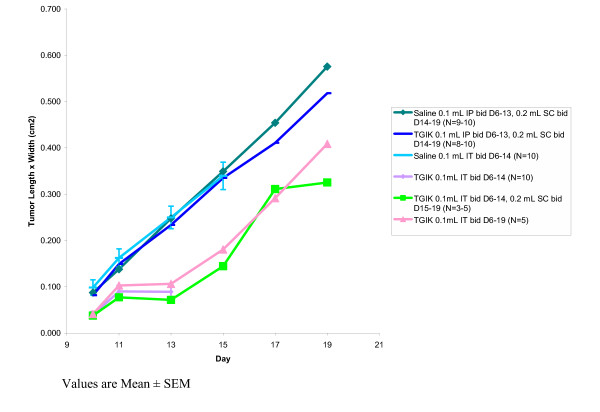
Antitumor activity against murine melanoma B16-F10 in C57BL/6 mice following TGIK administration via different routes of administration.

Five groups of C57BL/6 mice (ten mice per group) were injected subcutaneously on Day 1 with murine melanoma B16-F10 cells (1.8 × 10^6 ^cells) in the ventral aspect of the right hind limb. Injections with saline control and the TGIK formulation were begun on Day 6. Each milliliter of the TGIK formulation contained: insulin 3U, sodium thyroxine 50 μg, KCl 8 μEq, and glucose 50 mg. Tumor dimension (average length × average width) was determined on Days 10, 11, 13, 15, 17, and 19 and the results are indicated in Figure 1.

The results shown in Figure 1 demonstrate the antitumor efficacy of TGIK when administered by twice-daily intratumoral injection. Systemic administration (IP or SC) at these doses did not appear to offer any therapeutic benefit. The experimental design however, did not fully assess the possibility of a dose response relationship and consequently a potential benefit from larger doses administered systemically cannot be ruled out.

### Experiment 2

In order to determine whether the combination of all four ingredients of the TGIK formulation was required, and specifically to rule out the possibility that the antitumor effects observed in Experiment 1 were due only to an irritant effect of potassium, an experiment was conducted using the B16-F10 melanoma line in C57BL/6 mice in which the complete TGIK formulation was compared against GK and TGK as well as a saline control.

**Figure 2:**
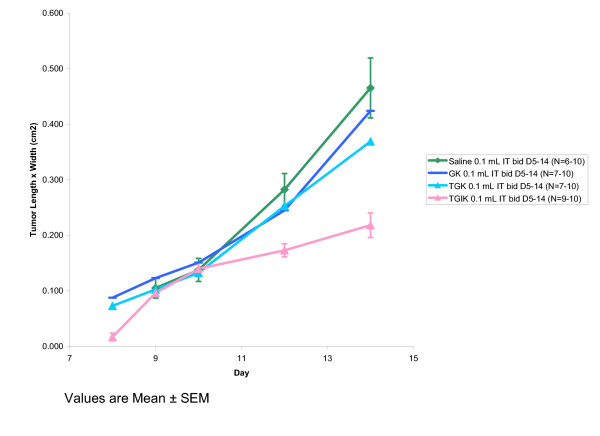
Antitumor activity against murine melanoma B16-F10 in C57BL/6 mice following administration of TGIK in comparison to incomplete formulations.

The results shown in Figure 2 demonstrate the activity of intratumoral TGIK and the finding that the formulation is rendered ineffective by removal of insulin. Consequently, this experiment demonstrates that the antitumor activity of TGIK is not due to an irritant effect from KCl alone.

**Figure 3:**
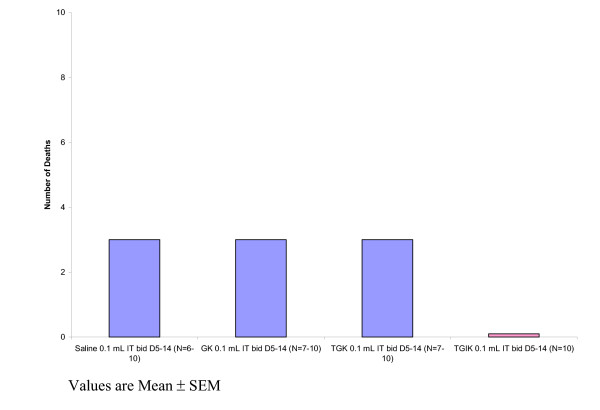
Mortality resulting from murine melanoma B16-F10 in C57BL/6 mice following administration of TGIK in comparison to incomplete formulations.

Figure 3 shows an incidental finding of this study. There was a reduction in mortality in the TGIK group relative to the other treatments.

### Experiment 3

**Figure 4:**
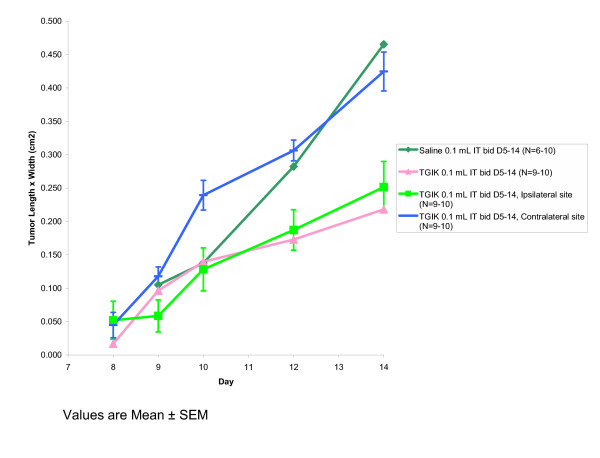
Antitumor activity against murine melanoma B16-F10 in C57BL/6 mice following administration of TGIK into the tumor site in comparison to growth in the contralateral tumor site.

Two additional groups of mice were injected with tumor cells in both hind limbs with only one hindlimb receiving subsequent TGIK injections to assess whether there was any effect on the contralateral tumor. The results are indicated in Figure 4.

Figure 4. In contrast to the potent antitumor activity of the formulation injected directly into the tumor site, there was no evidence of effect on the contralateral tumor site.

### Experiment 4

**Figure 5:**
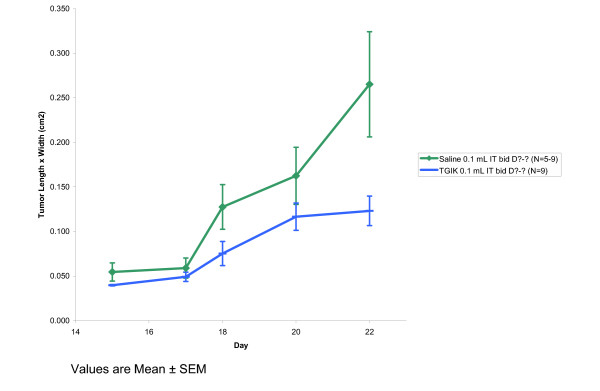
Antitumor activity against murine colon carcinoma CT26 in Balb/C mice following administration of TGIK.

These experiments were conducted in an analogous fashion to Experiment 1 except that the tumor line studied was the CT26 colon carcinoma line, the mouse model was the BALB/c mouse, and the tumor injection was of 50–100,000 cells per injection. Only the IT route of TGIK administration was evaluated. Because the tumors formed were more indurated, the mice were shaved to improve measurement determinations. The results of this experiment are presented in Figure 5.

As can be seen from Figure 5, TGIK is active against murine colon carcinoma cells, although the effect is somewhat more modest than its demonstrated activity against murine melanoma cells, perhaps a consequence of the slower growth rate of the colon carcinoma cell line. The colon carcinoma tumors tended to be more nodular and grow into deeper tissues making the tumor size more difficult to assess.

### Conclusions from the Preclinical Pharmacology Controlled Experiments

• The purpose for creating this model was to develop a more effective treatment for cancer. The aim of this series of controlled experiments was to prove that the cocktail would have anti-cancer activity. We realize these experiments do not prove the mechanism was immunological. However, the data produced in these experiments and in the low-dose human trials described below strongly suggest that immunity is the mechanism. An *in vitro *study in which tumor cells are exposed to the hormone cocktail without lymphocytes present would help to settle the issue. Also, a trial with nude mice would give more credence to immunity as the effective agent if the tumor's growth in that animal is not retarded, but those studies are not feasible for us at this time.

#### However, we believe the following conclusions are justified

• TGIK demonstrates potent antitumor activity against murine cancer cell lines transplanted into murine models

• Insulin is a required component of the TGIK formulation

• At the doses and regimens studied, antitumor activity is mediated by a direct response within the tumor without evidence of a systemic response affecting distant sites

### Preliminary human trials

Early low-dose Phase I trials for Hill Medical, using one injection of long lasting insulin per day with other materials administered orally, produced large rises in the CD4/CD8 ratio, with one patient reaching 71:1. Levels for normal patients are 3:1, for cancer patients ca. 2:1 or lower, and for AIDS patients much lower. More trials, better controlled, with higher doses of all materials administered intravenously, and with frequent measurements of blood insulin are in the planning stage. It is of interest that a psychiatrist in the 1950s administered a modified insulin shock treatment to two depressed cancer patients and the patients' tumors disappeared [57].

## Discussion

Great progress has been made in understanding the factors that regulate immunity. Immunologists have identified cytokines that up- or down-regulate immune functions. Others have created effective vaccines. Yet vaccines cannot be created for many diseases. Attempts to stimulate the immune system with cytokines to attack tumors have been disappointing. The doses most effective are unacceptably toxic [58]. But just as dreams of stimulating the immune system to attack tumors or more effectively deal with pathogens seem to be fading, there comes news of the surprisingly beneficial effect of GIK in treating the critically ill. Already both the American College of Cardiology and the American Heart Association have recommended that intravenous GIK be given to patients with acute myocardial infarction, even though the mechanism is still controversial. Since GIK apparently provides no benefit for patients with heart failure [59], we think it unlikely that the major benefit comes from a direct action on the heart.

We have proposed that GIK provides benefit to the critically ill patient because it stimulates lymphocytes. As the adaptive phase intensifies, activated lymphocytes release cytokines (IL-4, Il-10) [60] that down-regulate inflammation. Because septic shock is still the most common cause of death in the Intensive Care Unit, is the 10^th ^leading cause of death overall, has increased 86% between 1979 and 1997, and costs $5–10 billion for treatment, an effective prophylactic or treatment is urgently needed. We propose that GIK (and TGIK) are capable of protecting the patient against what are probably hospital-acquired infectious agents.

Van den Berghe also reported a reduction in critical illness polyneuropathy among her patients receiving GIK [61]. That syndrome is more likely due to a pre-existing, smoldering infection by an unidentified pathogen. Flare-ups of chronic, often unperceived, infections when a patient is immune-compromised as from the stress of surgery or serious injury are common. Inflammation is being implicated in more and more diseases, from Alzheimer's [62] to cancer, [63] and to autoimmune diseases such as lupus and diabetes [64]. But we propose that if patients threatened with polyneuropathy benefit from GIK, it is not because GIK reduces inflammation *per se*. It is due to GIK stimulating lymphocytes to efficiently remove the offending pathogen and to down-regulate inflammation with appropriate cytokines. In a recent discussion of the ideal treatment for Chlamydia, Ojcius, Darville and Bavoil have proposed that any intervention should evoke just enough inflammation to help the body's other immune defenses eliminate the bacteria [65]. In our model that is what happens when high doses of GIK are administered intravenously for a period of several hours. Reactivated lymphocytes attack pathogens and release cytokines to reduce harmful inflammation. If GIK prevented or ameliorated polyneuropathy, it might do the same for other chronic infections or auto-immune diseases.

We propose that chronic diseases like AIDS and atherosclerosis and amyotrophic lateral sclerosis (ALS) are caused by an *inadequate *immune response with little involvement by lymphocytes. We also suggest that auto-immune diseases are not due to an overly zealous attack by lymphocytes but to a continual, ineffective and destructive defense by inflammatory cells.

It is known that the development of many auto-immune diseases (e.g. insulin dependent diabetes mellitus (IDDM) [66], rheumatoid arthritis [67], Reiter's syndrome [68], Guillam-Barre Syndrome (GBS) [69], multiple sclerosis (MS) [70]) is preceded by a viral or bacterial infection or a vaccination. The course of these diseases is more like that of a chronic inflammation. Rheumatoid arthritis is an unrelenting disease that can continue for decades, and while "T cells are a prominent component of the inflammatory infiltrate in the rheumatoid synovium,... the more striking observation is the general paucity of T-cell-derived cytokines in the synovial tissue. In contrast, there is a wide range of readily detectable macrophage-derived products, including proinflammatory cytokines such as tumor necrosis factor-α and interleukin-1, that can activate synovial fibroblasts and other cells to produce matrix metalloproteinases involved in the degradation of cartilage" [71]. As Dinarello and Moldawer have said "...there is now growing recognition that persistent activation of the innate immune system occurs in a variety of autoimmune diseases, including rheumatoid arthritis. This prolonged activation leads to the constitutional complaints, metabolic abnormalities, and the destruction and remodeling of tissues experienced by patients with chronic and uncontrolled progressive diseases" [72].

We further propose that both chronic infections and many autoimmune diseases occur because of Antigenic Competition. It has long been known that if a patient is fighting one pathogen, infection by a second meets little resistance. To pathogen #2, there most likely will be an automatic, inflammatory response with phagocytosis of pathogen #2 by dendritic cells and tissue macrophages followed by presentation of antigen to lymphocytes. In our model there even may be minimal proliferation of lymphocyte clones, but those cells will be unable to mount an effective attack on the second pathogen. The inflammatory attack will cause some destruction of pathogens but also damage surrounding tissues. Fibroblasts may attempt to contain the infection by erecting fibrin barriers. But if the pathogen is multiplying more rapidly than the inflammatory attack, the infection will become chronic. Such an inflammation can go on for months, even years if lymphocytes are not activated to destroy pathogens.

In short, because of Antigenic Competition, the body can mount only one adaptive response at a time. Besedovsky and colleagues proposed that the phenomenon is caused by the increased level of corticosteroids induced by the first antigen [73]. If cortisol increases *after *the lymphocyte has already been stimulated by antigen, it will have no effect on the lymphocyte at physiological levels. But if cortisol rises *before *the lymphocyte is presented with antigen, the cell will be unable to respond. Also, it has been shown that "...CD8 lymphocytes after 4 hours of hyperinsulinemia in the normal subjects... had a sharp reduction in insulin-supported lymphocyte mediated cytotoxicity" [74]. A lymphocyte cannot respond if levels of insulin are high *before *it is challenged by an antigen.

So we proposed that the effect of high levels of cortisol and of insulin in the blood at the time of the second challenge is that the clone of lymphocytes that would ordinarily attack pathogen #2 are rendered helpless. We propose that even after infection #1 is resolved, the paralysis of clone #2 will often continue. It cannot activate without high levels of insulin for a prolonged period. Insulin will ordinarily rise only in response to another infection. But that is preceded by another surge of cortisol, which will continue the suppression of clone #2. However, in all cases of local inflammation (e.g. Pancreas, joints, myelin), there will be some activity by lymphocytes, both cellular and humoral. For acetylcholine, released from endings of cholinergic nerves, has much the same effect of enhancing the ability of cytotoxic lymphocytes to injure target cells [75]. The teleological benefit is that the body can send lymphocytes into a lesion to finish the killing of pathogens without having to mount a full scale systemic attack involving insulin. It seems unlikely, however, that the few infiltrating lymphocytes could fully meet the challenge presented to it by a disease such as rheumatoid arthritis.

We also suggest that if pathogen #2 is not contained in a local site but becomes systemic, it is likely that one of two things will happen. If the pathogen is virulent, sepsis will develop. The infection will rage uncontained, defended against only by the innate limb of the immune system, which, under such circumstances may itself be destructive. If the pathogen is a bacterium susceptible to antibiotics, the patient may be saved. Or, if the pathogen is less virulent, it may lodge in various tissues, only emerging at times of reduced immunity. It will produce shingles or attack skin or even organs, as in SLE or scleroderma.

Thus, in our model there are two circumstances in which the body cannot mount an effective adaptive immune response. The first is when the body abandons all effort to rid itself of pathogens and turns its energies to healing, as in the critical care setting. The second is Antigenic Competition.

We suggest that the only cure for lingering infections such as atherosclerosis, HIV or tuberculosis or for some auto-immune diseases, is infusion by GIK or TGIK to achieve levels of insulin that mimic those produced during an infection and for a long enough time for lymphocyte clones to fully proliferate and destroy the pathogen.

Unfortunately, it is likely that only studies with humans would conclusively prove or disprove this hypothesis. Animal models are of limited value in many of these diseases. Yet human experiments would be unacceptably dangerous. If conventional thought concerning autoimmune diseases is correct, the patient's condition would worsen, perhaps catastrophically.

However, it is possible that such studies have already, inadvertently, been conducted. Surely, some of the hundreds of patients who have been treated with high dose, long duration GIK in the critical care setting must have had Parkinson's or MS or ALS or Alzheimer's or Chlamydia or SLE or rheumatoid arthritis or GBS or scleroderma or atherosclerosis or tuberculosis or AIDS in addition to the acute condition that caused their hospitalization. What were the results for such patients? Was the condition ameliorated or exacerbated or did it remain unchanged? Follow-up studies of these patients could be helpful.

Before the possible full benefits of GIK can be assessed, questions of correct dosage, method of administration and duration of treatment must be settled. Treating a patient for 20 minutes [76], or even for a few hours, especially with low doses, would have little effect on immunity. More time is needed for full proliferation of activated lymphocyte clones. As Das has observed "Studies in which higher concentrations of insulin were used showed better results than did those studies that employed a lesser dose" [77]. We propose that GIK should be administered continuously and intravenously in whatever doses will maintain blood insulin levels at 35 ± 5 μU/ml for 48 to 96 hours to produce maximal benefit. In order to reach that level it may be necessary to adjust the dosage of insulin to each patient, but it is likely that insulin in the range of .1 to .15 U/kg/hr for non-diabetic patients should achieve this target level [78]. The patient must also receive enough glucose and potassium to avoid hypoglycemia and hypokalemia. Low doses of thyroid may be added to achieve maximum effect. Future researchers can contribute to the data base if they will perform pre-prandial testing of serum insulin and CD4/CD8 levels before, during, after treatment. Only studies with human patients can establish correct doses, duration of treatment and method of administration, but one of the advantages of GIK is that it is not a new drug. Clinicians are familiar with the signs of toxicity and counter-measures. The work of Van den Berghe and Krinsley show that can be done safely if patients are carefully monitored.

While van der Horst, *et al*. are correct that conclusive evidence GIK has a positive effect on sepsis is lacking [79], our work and that of others in a different setting are indicative of the importance of more research. For example, in 1985 Kowli, *et al*. reported that when they gave insulin in significant amounts to surgical patients, the infection rate was significantly lower than in controls and infection-related mortality was also reduced [80]. Also, if our experience with the increase in CD4 cells after treatment with low-dose TGIK could be reproduced, GIK may prove helpful in the treatment of AIDS.

The significance of the mounting evidence from GIK studies and the oncology studies cited above is obvious. For the first time physicians may be able not only to *reduce *inappropriate inflammatory and immune reactions, as with glucocorticoids, but also to *enhance *lymphocytic action to destroy pathogens and tumors without the use of toxic cytokines. It is, therefore, important that more research be devoted to establishing the mechanism and optimum dose and duration of treatment of GIK. Clinicians are already engaged in seeking that mechanism and the parameters for treatment. But immunologists have special knowledge that would be helpful in exploiting this important discovery.

## Competing interests

AFH holds multiple domestic and foreign patents on the use of TGIK and GIK for stimulating immunity and treating cancer. DBW and WJP have no competing interests.

## Authors' contributions

AFH conceived the model of immunity and the use of TGIK and GIK for treating cancer.

DBW designed and conducted the studies with mice and provided helpful advice on human trials.

WJP wrote the report on mice studies and is designing the protocol for a new trial of TGIK in humans.
